# Enabling simultaneous valorization of tannery effluent and waste plastic via sustainable preparation of Cr-BDC MOFs for water adsorption

**DOI:** 10.1038/s41598-023-41840-9

**Published:** 2023-09-05

**Authors:** Achraf Delhali, Ayalew H. Assen, Aminat Mohammed, Karim Adil, Youssef Belmabkhout

**Affiliations:** 1 Applied Chemistry and Engineering Research Centre of Excellence (ACER CoE), Mohammed VI Polytechnic University (UM6P), Lot 660 - Hay Moulay Rachid, 43150 Ben Guerir, Morocco; 2 Department of Chemistry, College of Natural Science, Wollo University, Dessie, Ethiopia; 3 Institut des Molécules et des Matériaux du Mans, Le Mans Université, Avenue Olivier Messiaen, 72085 Le Mans Cedex, France

**Keywords:** Chemistry, Materials science

## Abstract

Advanced materials undergo a complex and lengthy process of maturation for scaling up and deployment, mainly due to the high cost of their precursors. Therefore, it is highly desirable to fabricate highly valuable advanced porous solid-state materials, with proven applicability, by sustainably combining organic and inorganic waste materials as precursors. This study successfully demonstrates the preparation of Cr-terephthalate Metal–Organic Frameworks (Cr-BDC MOFs) by combining metal salt and organic linker extracted from tannery effluent and waste plastic bottles. The waste from tanneries was used as the source of Cr(III), while terephthalic acid was obtained from the alkaline hydrolysis of plastic bottles. Appropriate extraction and assembly processes led to the functional Cr-BDC MOFs, MIL-101(Cr) and MIL-53(Cr). The prepared MOFs showed similar properties (surface area, hydrolytic and thermal stability, and water adsorption performance) to similar MOFs synthesized from pure commercial-grade precursors, as confirmed by N_2_ sorption, XRD, TGA, and water adsorption experiments. The advancements made in this study represent significant progress in overcoming the bottleneck of MOF production cost efficiency via applying sustainability principles and pave the way for easy scaling-up and maturation of MOF-based processes, for air dehumidification and water harvesting as a case study.

## Introduction

Metal–organic frameworks (MOFs) are a recent type of porous material that has been remarkably effective in meeting the demand for functional solid-state materials that can be tuned to specific applications^1^. Over the last 2 decades, the number of crystal structures and applications of MOFs has grown substantially. Indeed, MOFs are promising materials for energy and environmental sustainability issues, and have already demonstrated great potential for catalysis^2^, chemical sensing^3^, gas storage and separation^4–7^, batteries^8^, and more^9,10^. MOFs' success in different applications is due to their structural tunability, high porosity, large surface area, and the versatility of the organic and inorganic precursors required for their assembly^11^. As an example, MOFs are highly effective in water harvesting and other water-sorption-related applications due to their exceptional tunability^12^. More than 97% of the planet's water is saltwater, and only 0.5% is available for human and ecological processes, with the majority being in highly stressed regions. On the other hand, the atmosphere contains a significant amount of clean water that can replenish the water basin^13^. As a result, water harvesting from the atmosphere has become a crucial research area to address water scarcity, especially in arid and semiarid regions. An ideal water harvesting material should have a suitable moisture adsorption–desorption profile, enabling high water productivity with minimal energy consumption and long-term operation^14^.

MOFs are a type of porous material with great potential for efficiently capturing water^15^. As an example, MIL-101(Cr) is a promising MOF for water-related applications^14^. However, their production at a large scale is hindered by the high cost of the materials used to make them. To address this issue, researchers have developed a solution using waste-based precursors to assemble functional MOFs, which could help make the production of these materials more sustainable^16^. Recent advances include the use of commercial metal sources and terephthalic acid obtained from recycled PET bottles to make MOFs such as MOF-5 (Zn-BDC)^17^, MIL-53(Al-BDC) or MIL-47(V-BDC)^18–20^, MIL-101(Cr-BDC)^19,21^, and UiO-66 (Zr-BDC)^22^. Recycled metal ions (V, Al, Cu, Ca, etc.) combined with commercially obtained organic ligands have also been used to assemble various MOFs^23–27^. Although MOFs made from industrial waste have been synthesized using either inorganic or organic components, a more sustainable approach using both metal salt and organic linkers from waste is underexplored^28–30^.

The waste chemicals released by the leather industry are a significant potential source of materials for MOFs^31^. Africa has a large number of livestock, which are often used for producing leather products, resulting in numerous tanneries being established in the region^32^. Despite the economic benefits of the growing leather industry, several environmental challenges need to be addressed. The use of toxic heavy metals like chromium as a tanning agent and the high cost of wastewater treatment has led to many tanneries discharging their waste directly into nearby rivers or other water bodies, which has severe environmental consequences. One potential solution is to use the effluent as a raw material for creating advanced chromium-based MOFs. Cr-MOFs, like MIL-53, MIL-100, and MIL-101, have exhibited significant potential for a range of applications because of their high moisture stability, good thermal stability, large pore volume, surface area, and the existence of unsaturated chromium sites^33–35^. The use of tannery effluent as a source of chromium for MOF synthesis is yet to be investigated.

Another major source of environmental pollution is waste plastic bottles made of polyethylene terephthalate (PET). Due to its low cost and durability, plastic has become ubiquitous in modern life. Every year, billions of PET bottles are used globally, with the number continuing to rise. For example, in 2018 alone, 8.3 billion metric tons of bottled beverages were produced, with an annual increase of 5% (185 million tons)^36^. This widespread acceptance of plastic, while affordable and efficient, poses a fundamental dilemma with the significant rise in plastic trash and its consequential impact on marine habitats. Currently, the plastic recycling industry is still modest, and PET plastic waste landfilling has led to serious environmental problems. The terephthalic acid ingredient of PET plastic bottles, a useful reactant for MOF synthesis, is also being wasted. PET is a condensation polymer of terephthalic acid (1,4-benzene dicarboxylic acid or H_2_BDC) and ethylene glycol, containing over 85wt% hydrolysable terephthalic acid in its ester form^37^. To address this issue, we were inspired by previous works that converted PET plastic into terephthalic acid and used it as an organic building block^18^. Our strategy involved using waste PET bottles as a source of H_2_BDC linker to synthesize Cr-BDC MOFs. MIL-53(Cr) or [Cr(OH)(BDC)]·*x*(solvent) is a 3D MOF comprising corner-sharing chains of CrO_4_(OH)_2_ octahedra interconnected by BDC linkers^38^. On the other hand, MIL-101(Cr) MOF, [Cr_3_(O)(OH/F)(BDC)_3_(H_2_O)_2_]·*x*(solvent), is built using BDC linkers and trimeric Cr(III) clusters generated in situ^39^. The MIL-101(Cr) framework structure contains mesoporous supertetrahedral cages formed by linking four trimeric clusters as vertices and six BDC linkers as edges. Cr-based MOFs derived from waste precursors, such as PET-derived terephthalates and commercial chromium salts, have been explored in only a few published papers^21,40–42^.

**Figure 1:**
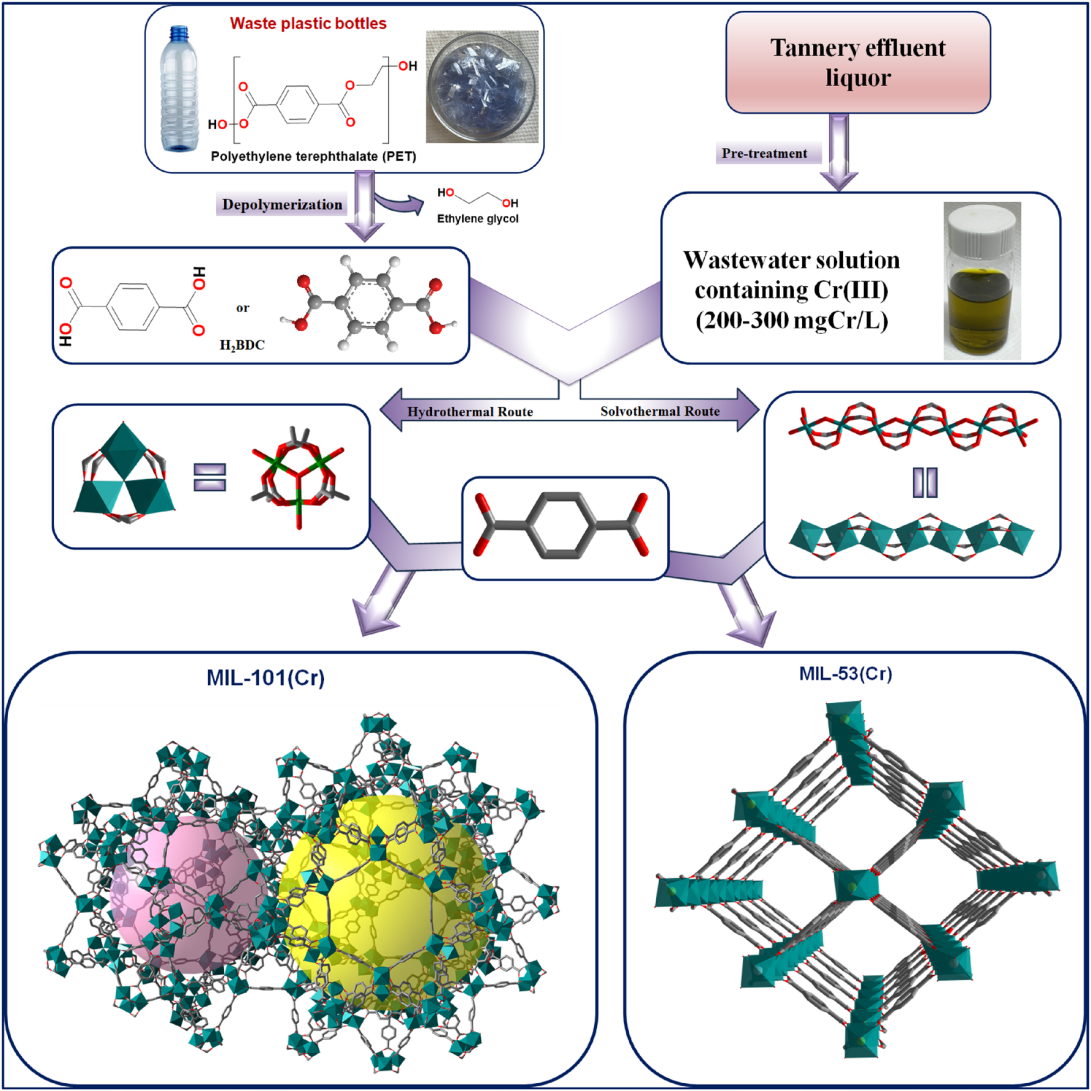
Schematic showing the assembly of the Cr-BDC MOFs, MIL-101 and MIL-53, from tannery effluent as a Cr source and terephthalic acid from the depolymerization of PET bottles.

In this study, we present a method for synthesizing Cr-BDC MOFs, namely MIL-101(Cr) and MIL-53(Cr), using (1) Cr(III) precursors derived from tannery effluent and (2) terephthalic acid obtained from hydrolyzing waste plastic bottles. A schematic illustrating the assembly of MIL-53(Cr) and MIL-101(Cr) from the target inorganic and organic waste precursors is depicted in Fig. 1. Finally, we compared the properties of the prepared MOFs with those of similar MOFs synthesized using analytical grade precursors.

## Methods

### General synthesis strategy

**Figure 2:**
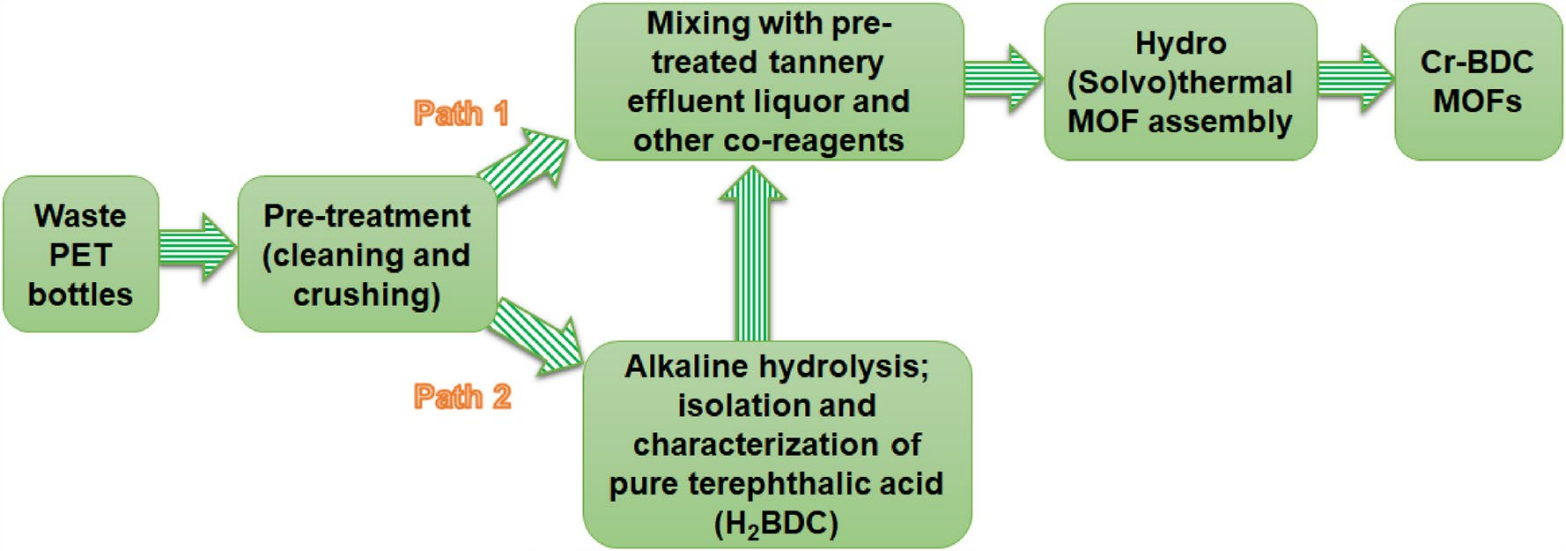
Schematic illustration of the one-pot and two-step MOF assembly processes used in the research to prepare Cr-BDC MOFs from waste PET bottles and tannery effluent. The tannery effluent is pretreated by filtration to remove insoluble suspended solid impurities, followed by evaporation/heating to increase the chromium concentration by about ten-fold.

The MOFs were created using either a one-pot or two-step solvothermal/hydrothermal reaction process. In the one-pot method, pre-treated tannery effluent and PET flakes were combined under appropriate solvothermal conditions. The stepwise process involved the hydrolysis of the PET bottles to extract pure terephthalic acid before the MOF synthesis. This was necessary to have better control of crystallinity. For more information on the hydrolysis process of the waste PET bottles, please refer to the supplementary material. A simplified flowchart outlining the experimental steps used can be found in Fig. 2.

### Cr-BDC MOFs synthesis

To prepare the targeted Cr-BDC MOFs, the brown-colored tannery effluent liquor, collected from the tannery's chrome-tanning pond, was filtered to remove some insoluble impurities before use. The MOF preparation was then completed using a solvothermal/hydrothermal synthesis approach, with proper trial and adjustment of reaction times. The MOF synthesis from waste-based precursors was achieved in two ways: one involved using waste precursors directly in a one-step process, and the other involved extracting the required terephthalic acid precursor before MOF preparation in a two-step process. To compare the properties of the MOFs assembled from waste precursors, similar MOFs were prepared using pure analytical grade precursors (terephthalic acid and chromium salt) obtained from commercial vendors. Details of the MOF synthesis can be found in the supplementary material.

### Characterization

The amount of total dissolved chromium in the tannery effluent liquor was estimated using Atomic Absorption Spectrophotometer (AAS). The extraction of pure terephthalic acid from PET bottles was confirmed through Fourier-transform infrared spectroscopy (FTIR) and NMR spectroscopy analyses. The MOFs phases were confirmed by powder X-ray diffraction (PXRD) and thermogravimetric analysis (TGA) characterizations, while the porosity of the obtained Cr-MOFs was confirmed through N_2_ adsorption experiments at cryogenic conditions. The elemental mapping of the waste-based MIL-101(Cr) sample was analyzed by SEM–EDS characterization. The water adsorption of MIL-101(Cr) was examined through H_2_O adsorption measurements. We used a Quantachrome Vstar1 vapor sorption analyzer to collect water vapor adsorption isotherms. In the experiment, we degassed approximately 50 mg of the sample in situ at 150 °C under a dynamic vacuum for 12 h. Activated (guest-free) samples were utilized for corresponding isotherm measurements, while the manifold temperature was kept constant at 110 °C throughout the procedure. More information on the characterization techniques can be found in the supplementary material.

## Results and discussion

### MIL-101(Cr) synthesis from tannery effluent and waste PET bottles

The two-step hydrothermal reaction process was used to create MIL-101(Cr), which is a typical Cr-BDC MOF. Before the MOF synthesis, atomic absorption spectroscopy (AAS) was used to estimate the total dissolved chromium concentration in the tannery effluent. The samples used gave slightly different values ranging from 228.4 to 294.3 mg/L, with an overall mean value of 263.7 ± 28.2 mg Cr/L (Table S1). The MOF synthesis was then carried out using the concentrated tannery effluent liquor that had been evaporated to increase its concentration ten times. The pure terephthalic acid ligand was also isolated from pre-treated plastic bottles using alkaline hydrolysis in ethylene glycol solvent for the MOF assembly. Before use in MOF preparation, the extracted organic product was dried and analyzed by FTIR and NMR spectroscopy. The FT-IR analysis (Figure S1) of the extracted H_2_BDC from PET showed a spectrum comparable to that of the commercial H_2_BDC (Sigma-Aldrich), providing additional evidence to support the successful depolymerization of PET bottles into terephthalic acid. The band at around 1683 cm^−1^ corresponds to the stretching of the C=O groups in the carboxylic acid. Characteristic peaks of O–C=O stretching vibrations were observed at 880 cm^−1^, and the other bands at 715–725 cm^−1^ can be assigned to the aromatic rings^21,22,43,44^. NMR analyses (Figure S2) further confirmed the formation of pure H_2_BDC linker by alkaline hydrolysis of plastic bottles. In the ^1^H NMR spectrum, the peak at 8.02 ppm corresponds to the four aromatic protons of the terephthalic acid ligand, while the peak at 2.50 ppm corresponds to the DMSO-d_6_ solvent protons. The carboxylic acid protons of the linker are visible at 13.22 ppm. ^13^C NMR analysis also confirmed the formation of pure BDC linker by alkaline hydrolysis of plastic bottles. The peak at 167 ppm corresponds to the carboxylate carbons of the BDC linker, and the peaks at 134 and 129 ppm correspond to the quaternary and CH carbons, respectively.

**Figure 3:**
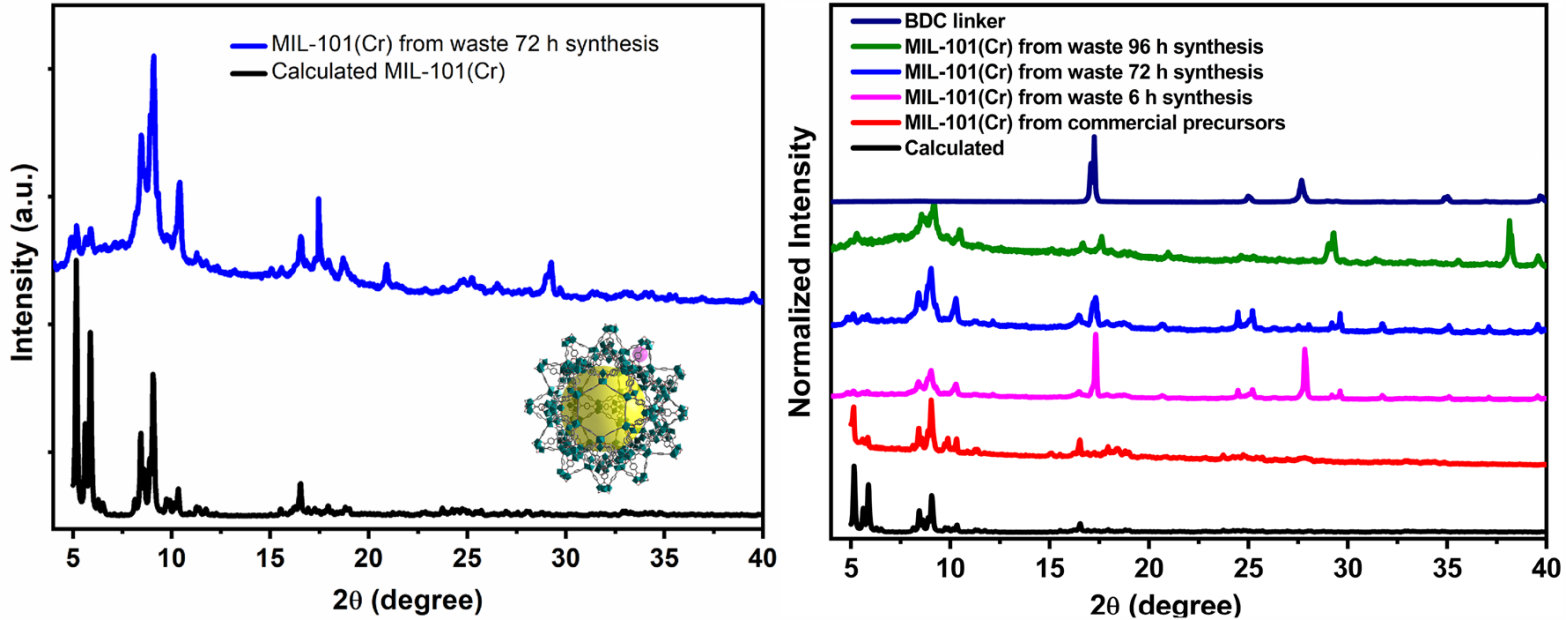
Comparison of the obtained experimental PXRD patterns with that of calculated MIL-101(Cr) MOFs (left) and experimental PXRD patterns of solids obtained from wastes under different reaction times during the target MIL-101 synthesis (right).

Once the isolation of H_2_BDC from plastic bottles was confirmed, the target MOF was prepared using hydrothermal synthesis by heating solutions containing tannery effluent liquor and terephthalic acid extracted from PET bottles. In a typical reaction, 170 mg of H_2_BDC linker (isolated from waste PET bottle by hydrolysis) was mixed with 15 mL of tannery effluent liquor (concentrated by about tenfold by heating 150 mL effluent) in a Teflon-lined stainless-steel autoclave. The hydrothermal reaction at 180 °C led to the formation of green powder after 72 h reaction time. The as-synthesized material was activated by solvent exchange with methanol followed by direct evacuation of pores under vacuum. The obtained green powder was structurally characterized using powder XRD, revealing the formation of the MIL-101 framework with no phase impurity. This was confirmed by the similarities between simulated and experimental PXRD patterns (Fig. 3). Shorter reaction times (< 12 h) also resulted in MIL-101 phases but were dominated by the unreacted crystalline phase of terephthalic acid (Fig. 3 right). Elongating the reaction time for more than 3 days gave MIL-101 phase but with oxide impurity. The researchers also attempted a one-pot solvothermal reaction with tannery effluent liquor and PET flakes, but only amorphous products of unknown phases were obtained.

Besides the main component (Cr), chrome tanning powders and many re-tanning agents contain sodium sulfate that could later remain in the effluent. The common salt used to preserve hides and skin also contributes to some amounts of sodium and chloride ions in the effluent. Therefore, the major cations that could exist in the tannery waste from the tanning pond is sodium, while the major anions are sulfate and chloride^45^. Studies also showed the presence of other metal cations (Cu, Zn, Cd, Fe, etc.) in tannery effluents but only in trace amounts (< 100 ppm)^46^. In such concentration, the metal cations cannot compete to form M-BDC MOFs during the reactions of Cr-BDC MOF assembly. The anions are not expected to affect the reactions since chromium in both sulfate and chloride forms can form Cr-BDC MOFs. This assertion is substantiated by the EDS spectra obtained for the synthesized waste-based sample (Figure S6) which exhibit a substantial similarity to the chemical composition of the MIL-101(Cr) framework, [Cr_3_(O)(OH)(BDC)_3_(H_2_O)_2_]. The EDS-derived elemental mapping shows the presence of Cr, C, and O atoms with no other metal and nonmetal impurities from the waste.

### MIL-53(Cr) synthesis from tannery effluent and waste PET bottles

**Figure 4:**
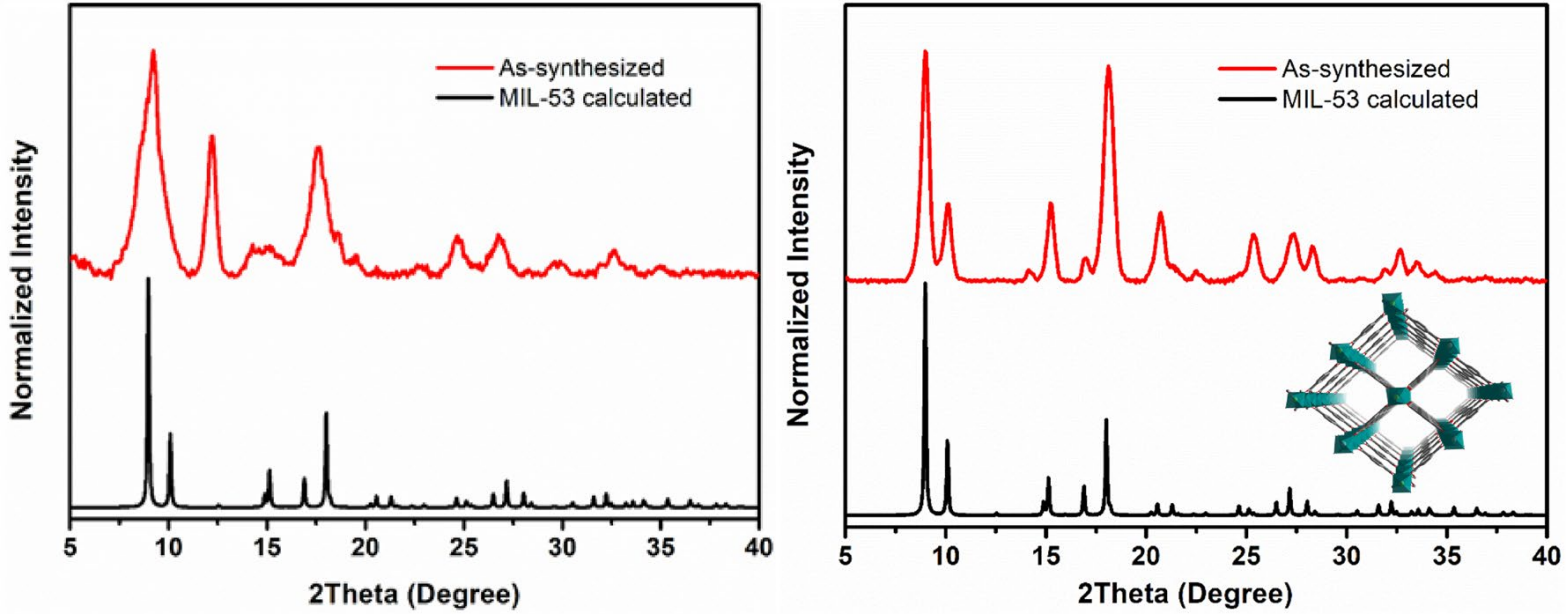
Comparison of experimental PXRD patterns of the waste-based MIL-53(Cr) MOFs assembled by one-pot (left) and two-step (right) synthesis routes with the calculated PXRD of MIL-53 framework.

The ability to transform two waste materials into MOFs in a single step was demonstrated in another typical Cr-MOF platform, MIL-53(Cr). The MOF synthesis process involved using the waste precursors directly, which means mixing unhydrolyzed PET with tannery effluent liquor. In the standard reaction, we took 100 mL of filtered wastewater, which was concentrated to about 10 mL. Then, we mixed the concentrated effluent with pre-treated plastic bottles that were cleaned, dried, and cut into small flakes. The reaction of these components in the presence of NaOH and a water/ethylene glycol solvent mixture under solvothermal conditions resulted in a light green powder. The obtained powder was analyzed by XRD, and the peaks were matched with the simulated XRD patterns of the MIL-53 phase (Fig. 4 left and Figure S4). Despite further attempts to improve the crystallinity of the MOF crystals, better results could not be achieved.

To improve the crystallinity of the MOF, a two-step procedure was used to better control the reaction conditions. Firstly, the tannery effluent liquor was filtered to remove solid impurities and concentrated, as in the one-pot procedure. Next, the pre-treated liquor was combined with pure H_2_BDC linker obtained from waste PET bottles and subjected to solvothermal reactions. Experimental PXRD patterns were then compared to calculated ones, deduced from the crystal structure of MIL-53 (Fig. 4 right), and it was confirmed that the MOF obtained from waste-derived precursors is MIL-53(Cr). The PXRD patterns revealed no other Cr-BDC MOF polymorph formations, indicating the bulk phase purity of the MOF. The calculated PXRD diagram was obtained from the crystal structure file of MIL-53(Cr) MOF previously reported in the literature^38^. The PXRD patterns of waste-derived MIL-53(Cr) also match correctly with the PXRDs of similar MOFs synthesized from commercial-grade chromium salt and terephthalic acid (Figure S3). Notably, the PXRD of the MOF obtained by the one-pot hydrothermal route has broader peaks than the similar samples obtained from commercial precursors and the two-step synthesis.

### Hydrolytic and thermal stability tests

Ensuring the stability of adsorbents under hydrothermal conditions is crucial for their effective use in water adsorption applications. Therefore, stability tests were conducted on the MOF samples produced from waste-derived Cr and BDC precursors using a two-step solvothermal synthesis. The hydrolytic stability of the synthesized MOFs when exposed to water was evaluated by immersing the MOF samples in water for 3 days, and the PXRD measurements were taken before and after the test. The results indicated no alteration in the crystallinity or phase changes, indicating the materials' excellent stability characteristics against moisture (Figure S5).

**Figure 5:**
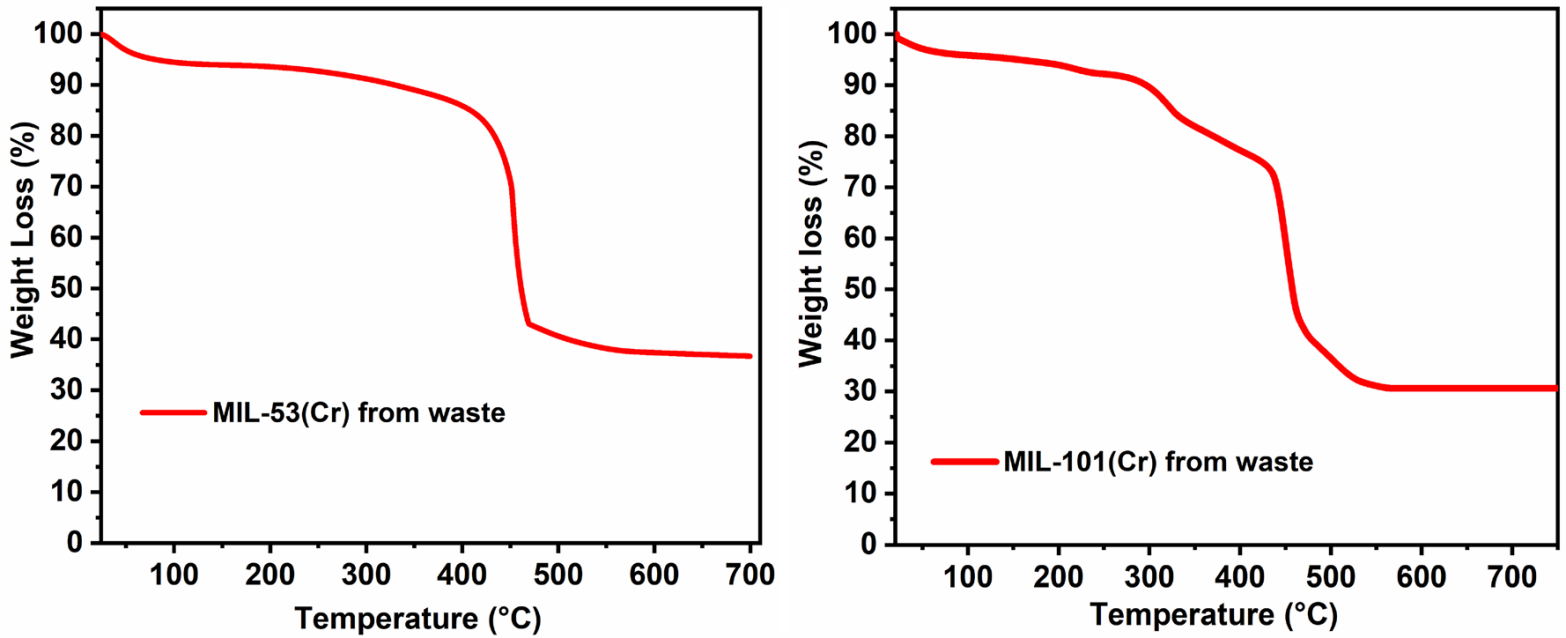
TGA traces for the MIL-53(Cr) (Left) and MIL-101(Cr) (Right) MOFs prepared from tannery effluent solution and terephthalic acid derived from the depolymerization of plastic bottles.

The MOFs' structural composition and thermal behavior were characterized by thermogravimetric (TGA) measurements (Fig. 5). For the TGA experiment, methanol-exchanged analogs of the MOF samples were used. The samples were dried in the open atmosphere and then under compressed air flow to remove traces of solvent on the surface. As shown in Fig. 5 (left), the experimental weight losses in the TGA analysis are in close agreement with the MIL-53(Cr) composition of [Cr(OH)(O_2_C-C_6_H_4_-CO_2_)]·*x*(solvent). A first loss of about 6 wt% was observed until ~ 150 °C, which can be attributed to residual non-coordinated solvent molecules. The frameworks degraded after 400 °C, and the organic ligand (~ 61%, calculated = 70.3% assuming guest-free framework) departed to form the corresponding oxide residue (~ 34%, calculated = 32.6% assuming the formation of 0.5 mol Cr_2_O_3_ from 1 mol of the MOF). The TGA analysis showed that the synthesized MOF samples have good thermal stability, similar to the MIL-53(Cr) MOF previously reported from pure commercial chromium and BDC precursors^38^. Additionally, the thermal property of the waste-derived MIL-101(Cr) sample was established by TGA measurements. The TGA analysis showed that the synthesized compound loses ~ 4.2% extra framework solvent up to almost 100 °C without further significant weight losses till 150 °C (Fig. 5 right). The weight loss in the 150–300 °C temperature range (~ 5.3%) could be due to the departure of the two coordinated water molecules of the framework which account 5% of the calculated composition in the framework formula, Cr_3_(O)OH(O_2_C-C_6_H_4_-CO_2_)_3_(H_2_O)_2_. Framework decomposition due to ligand departure occurs in the 300–550 °C range. The experimental weight losses due to ligand departure (~ 60.6%) and the residual weight % of chromium oxide (Cr_2_O_3_) in the TGA of the dry sample (~ 29.9%) are in close agreement with the calculated values from the expected formula of MIL-101(Cr) crystal structure (calculated for BDC = 68.6% from guest-free framework and expected Cr_2_O_3_ residue = 31.7% assuming the formation of 1.5 mol Cr_2_O_3_ from 1 mol of the MOF).

### Permanent porosity comparison of waste-derived and commercial reagent-based MOFs

After confirming that the samples were pure and stable, we used N_2_ adsorption experiments at 77 K to determine the surface area and pore volume of the MOFs we synthesized. Before conducting the N_2_ adsorption tests, we replaced the solvent in the materials with methanol for 3 days, refreshing the methanol solvent three times per day. The methanol-exchanged samples were then evacuated by heating them under a vacuum at 150 °C. The nitrogen sorption isotherms recorded at 77 K showed that the MIL-53(Cr) MOFs prepared using a one-pot hydrothermal route from waste-based precursors (Figure S7a), a two-step solvothermal synthesis from waste-derived precursors, and a one-pot hydrothermal reaction from commercial-grade precursors (Figure S7b) exhibit Type-I isotherms. This characteristic is consistent with microporous materials according to the IUPAC classification, and the BET surface areas of the MOFs were 451, 890, and 996 m^2^ g^−1^, respectively. The total micropore volumes from the nitrogen sorption study at P/P_0_ ~ 0.90 for the three MOF samples were 0.45, 0.55, and 0.62 cm^3^ g^−1^, respectively. The lower nitrogen adsorption uptake, i.e., the smaller pore volume, for the MOF isolated using the one-pot synthesis condition compared to the comparable adsorption capacities of the other samples could be due to the lower quality of the material from the one-pot synthesis, which is evident in its diffractogram. For the case of MIL-53 sample from waste obtained in the two-step procedure, the surface area and pore volume values are comparable to those reported in the literature for the same MOF.^47^ Both the two-step waste-derived and commercial precursor-based MIL-53(Cr) samples also revealed a uniform pore size distribution (PSD) (Figure S7c–d) with one type of pore that displays an average size centered around 7 Å, which is in good agreement with the one type of diamondoid channel of the MIL-53 framework.

**Figure 6:**
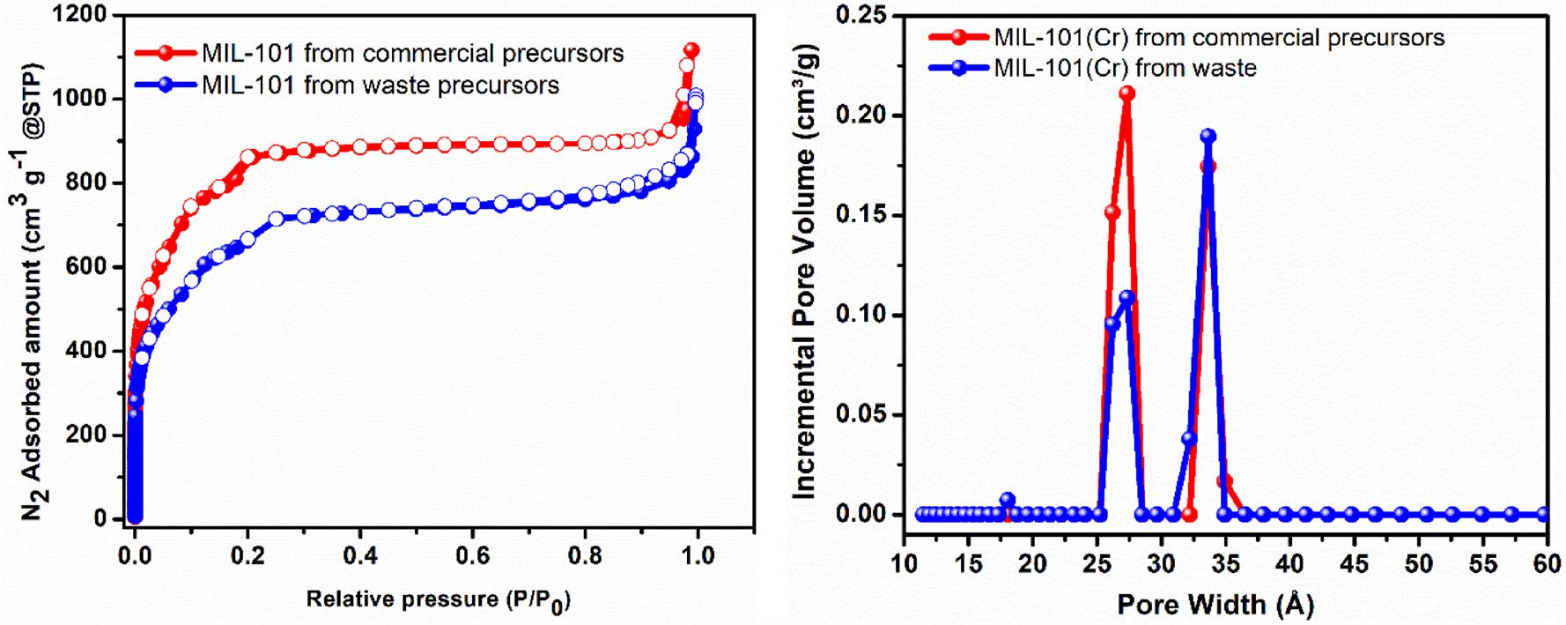
N_2_ adsorption isotherms (closed symbols: adsorption and open symbols: desorption) of MIL-101(Cr) synthesized from commercial precursors and waste by two-step synthesis (Left) and Pore size distribution analyses for the MOFs deduced from the corresponding N_2_ adsorption isotherms using DFT model (Right).

We also conducted N_2_ adsorption tests to verify the porosity and pore size of the prepared MIL-101 framework from waste and commercial precursors. The N_2_ isotherms of the MOFs recorded at 77 K exhibited the expected fully reversible isotherms of the mesoporous MIL-101 material (Fig. 6). The waste-based and commercial precursor-based MIL-101 samples exhibit BET surface areas of 2546 and 2849 m^2^ g^−1^, respectively. The total pore volumes at P/P_0_ = 0.95 from the N_2_ sorption study for waste- and commercial precursor-based samples are 1.29 and 1.43 cm^3^ g^−1^, respectively, which closely match the previously reported values for the same framework. The values obtained for the waste-based MIL-101 sample generally fall within the ranges previously reported for the commercial precursor-based MIL-101(Cr)^34^. Figure 6 also depicts the pore size distribution (PSD) curves of MIL-101(Cr) derived from waste and MIL-101(Cr) prepared using commercial precursors. We found that these materials had nearly identical pore structures. The determined PSDs deduced from the N_2_ sorption isotherms showed the expected mesopores with average sizes centered around 27 and 33 Å, consistent with the different pore size dimensions observed for the MIL-101(Cr) crystal structure^39^.

### Water adsorption study on waste-derived MIL-101(Cr) and its commercial precursor-based analog

**Figure 7:**
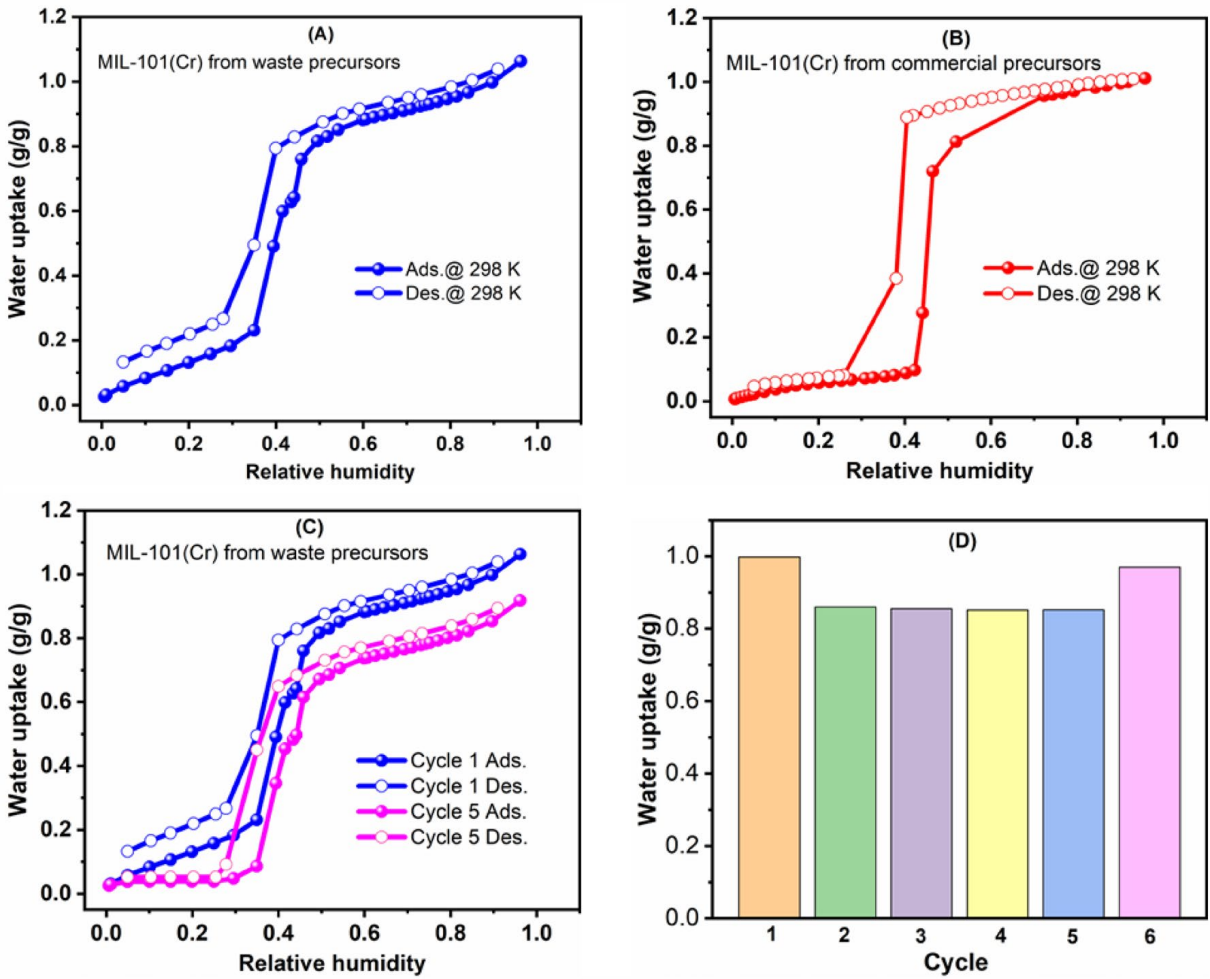
H_2_O adsorption isotherms collected at 298 K for waste-derived (**A**) and commercial precursor-based (**B**) MIL-101(Cr) MOFs; (**C**, **D**) Cyclic water adsorption isotherms for the 1st and 5th cycle (**C**) and water uptakes at 90% relative humidity (**D**) for the waste-derived MIL-101(Cr).

After conducting extensive tests to ensure the purity, hydrolytic stability, and porosity of the samples, we proceeded to test the water adsorption abilities of the MIL-101 Cr-MOFs synthesized from waste-derived materials. We found that the waste-derived MOF displayed a sigmoidal-shaped isotherm with a high adsorption capacity (~ 1.00 g g^−1^ at saturation) and a moderate hysteresis loop at RH ~ 0.4. These results were comparable to the adsorption profile and capacity of a similar MOF prepared from commercial precursors (Fig. 7A, B).

To confirm the materials' long-term reusability, we conducted cyclic water adsorption–desorption measurements on the waste-derived MIL-101. The material exhibited remarkable cyclic durability for six consecutive cycles with nearly constant water uptake after the first cycle (Fig. 7C, D). The lower uptake in the second cycle could be attributed to insufficient regeneration of strongly adsorbed water molecules occupying unsaturated chromium sites. Between each cycle of the 1st five cycles, the sample was kept under vacuum at ambient temperature for 4 h. This assertion was supported by the very close similarity of the 1st and 6th cycles water sorption data obtained after evacuating the material at higher degassing temperature prior to the adsorption measurements. Before the 6th cycle, regeneration was done at 120 °C for 4 h. Like the commercial precursor-based MOF, the waste-based MOF fulfills the requirements for air dehumidification and water harvesting, including good hydrolytic stability, steep uptake at a specific relative humidity (40% in this case), large working capacity for the requisite maximum delivery of water, minimal hysteresis, and high cycling durability. This makes the MOF a suitable candidate for combined air dehumidification and water harvesting in arid and semiarid regions with high relative humidity. Therefore, the strategy discussed here, which allows for leveraging waste as precursors to assemble Cr-based MOFs with unaltered properties, could alleviate the challenges associated with the large-scale synthesis of the MOF due to expensive commercial precursors while offering (1) circular economy solutions to tannery/plastic industries and (2) sustainable solution to water scarcity. In this work, we chose to demonstrate the water adsorption property of the waste-based MIL-101 framework. The inherent water stability and high water sorption capacity of MIL-101(Cr) have already been proven fundamental for water-sorption-based applications related to evaporative cooling, desalination, heat transformation, such as in adsorption chillers, and many more^15^. The water adsorption performance obtained for the MIL-101(Cr) isolated from waste organic and inorganic starting materials could create an opportunity for low-cost adsorbent synthesis and deployment in water-related industrial applications.

In this study, we utilized organic and inorganic waste precursors to create Cr-MOFs, MIL-101(Cr) and MIL-53(Cr) in a sustainable way, which contribute to circular economy endeavor and significantly reduces the production costs of scaled-up MOF fabrication. The cost of the synthesis reagents used to make the Cr-MOFs reported here, such as ethylene glycol ($0.6/kg) and NaOH ($0.02/g), are much lower compared to the costs associated with the conventional synthesis of Cr-MOFs, which require larger quantities of analytical grade chromium salts (chromium(III) nitrate nonahydrate or chromium(III) chloride hexahydrate, $0.20/g) and terephthalic acid ($0.05/g) to produce similar MOFs. Assuming 80% MOF yield, the cost of organic and inorganic precursors is estimated to be about $450 to produce 1 kg of MIL-101(Cr) from commercial reagents. Using tannery effluent and waste PET bottles saves approximately $400 when considering to the cost of ethylene glycol and NaOH required to produce the desired amount of terephthalic acid (~ $50). Although the operating expenses are similar in both waste-to-MOF and commercial precursor-to-MOF procedures, the waste-to-MOF synthesis route provides further process flexibility and cost reduction by allowing the fabrication of Cr-MOFs using wastewater instead of the expensive DMF or deionized water solvent. The approach described in this study is a potential pathway to sustainably produce MOFs at a low cost on a large scale. Although additional pre-treatment purification steps would be required, using tannery effluent and waste PET bottles as precursors significantly reduces the cost of Cr-MOF production.

## Conclusion

This work demonstrates the possibility of transforming two different waste-based raw materials into advanced adsorbents simultaneously. By using tannery effluent as a cheap source of chromium and waste plastic bottles as a source of terephthalate linker, sustainable precursors were obtained to create functional MOFs. The appropriate hydrothermal or solvothermal reactions allowed for the assembly of MIL-101(Cr) and MIL-53(Cr), two well-known functional MOFs. The purity, stability, and porosity of the MOFs were validated by PXRD and N_2_ sorption measurements. The waste-derived MOFs showed comparable BET surface areas (~ 890 m^2^ g^−1^ for MIL-53 and ~ 2550 m^2^ g^−1^ for MIL-101) and pore volumes (0.55 cm^3^ g^−1^ for MIL-53 and 1.30 cm^3^ g^−1^ for MIL-101) to those prepared from commercial-grade precursors. TGA experiments revealed the prepared MOFs' thermal stability up to 300–400 °C, while their hydrolytic stability was comparable to those prepared from pure commercial-grade precursors. The water adsorption performances of the MOFs from waste-derived precursors and commercial-grade reactants were also comparable, providing economic and environmental value. This approach can pave the way for creating various new generations of sustainable MOFs and encourages researchers to promote waste valorization and large-scale MOF synthesis. Further studies could help develop design principles for creating other functional MOFs from waste materials.

### Supplementary Information

Supplementary Information.

## Data Availability

The datasets generated and analysed during the current study are available from the corresponding author on reasonable request.
