# Supplementary material for: Enabling simultaneous valorization of tannery effluent and waste plastic via sustainable preparation of Cr-BDC MOFs for water adsorption
Source: Sci Rep. 2023 Sep 5;13:14653. doi: 10.1038/s41598-023-41840-9 (PMC10480428; doi:10.1038/s41598-023-41840-9)
Supplement: Supplementary file 1 — Supplementary Information. [file 41598_2023_41840_MOESM1_ESM.docx]

# **Supplementary Information**

# **Enabling simultaneous valorization of tannery effluent and waste plastic via sustainable preparation of Cr-BDC MOFs for water adsorption**

Achraf Delhali^a,c^, Ayalew H. Assen^b,^*, Aminat Mohammed^b^, Karim Adil^a,c,^*, Youssef Belmabkhout^a,^*

^a^Applied Chemistry and Engineering Research Centre of Excellence (ACER CoE), Mohammed VI Polytechnic University (UM6P), Lot 660 - Hay Moulay Rachid, 43150 Ben Guerir, Morocco

^b^Department of Chemistry, College of Natural Science, Wollo University, Dessie, Ethiopia

^c^Le Mans Université, Institut des Molécules et des Matériaux du Mans. Avenue Olivier Messiaen, 72085 Le Mans cedex, France

* Corresponding authors: Ayalew H. Assen; K. Adil; Y. Belmabkhout

E-mail: [ayalew.assen@um6p.ma](mailto:ayalew.assen@um6p.ma); [karim.adil@univ-lemans.fr](mailto:karim.adil@univ-lemans.fr); [youssef.belmabkhout@um6p.ma](mailto:youssef.belmabkhout@um6p.ma)

**1. Materials and Methods**

**1.1. Materials**

Tannery effluent liquor (collected from the wastewater ponds of the industry in Kombolcha, Ethiopia), Cr(NO₃)₃·9H_2_O (97%), sodium hydroxide (NaOH), sulfuric acid (H_2_SO_4_), hydrofluoric acid (HF) (48%), plastic bottles, terephthalic acid (benzene-1,4-dicarboxylic acid or H_2_BDC), organic solvents (ethylene glycol, N, N-dimethylformamide (DMF), methanol, ethanol, and acetone). Teflon-lined stainless-steel autoclaves (solvothermal synthesis reactors) were used to undertake hydrothermal syntheses. The concentration of dissolved chromium in the effluent ranges from 228.2 to 294.3 mg/L, with an overall mean value of 263.7 ± 28.2 mg Cr/L. The overall average value was calculated using the results of five different samples. Over the course of a month, five samples were collected, with approximately one sampling per week. Measurements were taken in triplicate for each sample, and an average value was calculated. Table S1 shows the mean value obtained for each sample as well as the overall mean value. However, the obtained Cr amount is too diluted to be used in the synthesis of the target MOFs. As a result, an additional step of concentration by evaporation/heating was used. Prior to using the tannery effluent for MOF synthesis, 100-150 mL of the effluent was transferred to a beaker. The effluent solution was then concentrated by evaporating the water until about 10-15 mL was left (concentrating it by about 10-fold). All non-waste-derived reagents were purchased from commercial sources and used without further purification. The water used in all the synthetic conditions is distilled unless otherwise stated.

Table S1. Cr concentration values (in mg L^-1^) for samples collected at different times.

| **Sample** | **Sample 1** | **Sample 2** | **Sample 3** | **Sample 4** | **Sample 5** | **Overall Average** |
| --- | --- | --- | --- | --- | --- | --- |
| **Mean value** | 281.2 ± 2.1 | 294.3 ± 1.5 | 274.7 ± 1.4 | 228.4 ± 1.1 | 239.8 ± 1.9 | 263.7 ± 28.2 |

**Note:** Most tanneries in Ethiopia use a chromium-based tanning method that involves several steps taking place in different treatment ponds. The first step, the beam house operation, entails soaking skins and hides in lime to facilitate the removal of hair. A bating process is then conducted using proteolytic enzymes to soften the skins and hides. In a subsequent step, the chrome tanning process converts collagen from the skins and hides into leather, producing the most hazardous wastewater containing a toxic chromium residue. The process is completed in the final step, i.e., finishing, which involves stretching, buffing, and/or drying the tanned product. The sewage system collects wastewater from all these steps. For our research, the waste effluent was taken from the chrome tanning pond to minimize the high concentration of calcium cation from the first step that could interfere with the Cr-MOF synthesis reactions. Calcium cations can easily interact with terephthalic acid forming various types of Ca-BDC MOFs.[^1^](#_ENREF_1)

**1.2. Hydrolysis of waste plastic bottles**

Plastic PET bottles (drinking water bottles) were collected from domestic waste. Labels and caps were removed, and the bottles were washed with distilled water, soap, and ethanol and then dried properly. The washed and dried plastic bottles were made into flakes by cutting the bottles into tiny pieces of around 1cm × 1cm. The waste PET bottles were used to form Cr-BDC MOFs in two ways: in situ hydrolysis of PET while undertaking MOF synthesis and alkaline hydrolysis of PET before MOF synthesis. In the second case, for the depolymerization of PET bottles, the procedure reported by Zhang et al.[^2^](#_ENREF_2) was adopted with slight modification. In a 500 mL round bottom flask attached to a reflux condenser, 10 g of PET flakes, 55 mL of ethylene glycol, and 12 g of NaOH were mixed. The solution was slowly stirred and heated to 180 °C under reflux. After 40 min, the plastic pieces gradually decomposed, and the solution turned milky white. After the completion of the reflux process, the round-bottom flask was cooled to room temperature. Subsequently, 300 mL of deionized water was added to the flask while stirring and then filtered under vacuum to remove the undecomposed plastic flakes. The filtered solution was stirred vigorously, and about 25 mL sulfuric acid (5 M in water) was added dropwise to form a white solid. The solution was kept stirring for 30 minutes. The precipitate was then separated by centrifugation, washed four times with deionized water, and dried at 90 °C overnight to obtain a white solid (6.8 g). The formation of pure terephthalic from the alkaline hydrolysis of waste PET bottles was confirmed by NMR and FTIR analyses.

**1.3. MIL-53(Cr) synthesis from waste precursors**

In the one-step MOF synthesis, MIL-53(Cr) was prepared under solvothermal conditions by heating wastewater solution containing Cr(III) precursor from tannery effluent solution (concentrated to 10 mL by heating) and flaked waste plastic bottles (200 mg) in the presence of ethylene glycol (5 mL) and NaOH (10 mg). After stirring in a beaker at 150 °C for an hour, the mixture was transferred into a Teflon-lined stainless-steel autoclave, and 0.1 mmol of HF was added. The autoclave was then tightly sealed and placed in a pre-heated oven at 220 °C for 3 days. A pale purple powder was then collected. The as-synthesized sample was washed with DMF and kept in DMF at 60 °C for 12 h. The powder was then immersed in methanol for three days, during which the methanol was replaced thrice daily.

In the two-step MIL-53(Cr) synthesis, the MOF samples were prepared hydrothermally by heating tannery effluent solution containing Cr(III) precursors (10 mL) concentrated by heating to evaporate the water, H_2_BDC linker (200 mg) isolated from the hydrolysis of waste plastic bottles, and HF (0.1 mmol). The reactant mixture was introduced in a Teflon-lined steel autoclave and then sonicated for about 30 min. It was then placed in an oven at 220 °C for 72 h. After three days in the oven, the autoclave was taken out and slowly cooled to room temperature. The as-synthesized light purple powder samples were washed with DMF and then exchanged with methanol. The solid was then dried at 150 °C under vacuum for 12 h to yield an activated/evacuated sample. About 92 mg of MIL-53(Cr) dry sample (approximately 78% yield, taking guest-free framework formulas for the theoretical yield calculation) was obtained from the reactions. The weight was measured after keeping the samples in a vacuum oven at 150 °C overnight. The percent yield calculation was made assuming the presence of an average Cr concentration (26.3 mg) in the starting pre-treated tannery effluent reactant (10 mL).

**1.4. MIL-101(Cr) synthesis from waste precursors**

The synthesis from waste was accomplished using the two-step procedure. In the typical procedure, the MOF was prepared hydrothermally by heating concentrated solutions containing Cr(III) precursors from tannery effluent (15 mL) and H_2_BDC linker (170 mg) isolated from the hydrolysis of waste plastic bottles. After introducing the precursors into a Teflon-lined stainless-steel autoclave, the mixture was sonicated for about 30 min and then placed in an oven set at 180 °C for 72 h. The as-synthesized green powder was washed with DMF and then soaked in methanol for three days refreshing the solvent three times a day. The solid was then dried at 150 °C under vacuum for 12 h to yield a guest-free (evacuated) sample. About 87 mg of MIL-101(Cr) dry sample (~72% yield, taking guest-free framework formulas for the theoretical yield calculation) was obtained from the reactions. The weight was measured after keeping the samples in a vacuum oven at 150 °C overnight. Like that of MIL-53, the percent yield calculation was made assuming the presence of an average Cr concentration in the starting pre-treated tannery effluent reactant.

**1.5. MIL-53(Cr) synthesis from commercial-grade precursors**

MIL-53(Cr) from the commercial precursors was prepared following the literature procedure.[^3^](#_ENREF_3) Cr(NO_3_)_3_·9H_2_O (400 mg, 1.0 mmol), terephthalic acid (167 mg, 1.0 mmol), and HF (1.0 mmol) were mixed in 15 ml deionized water and then sonicated for 15 min. The mixture was placed in a Teflon-lined autoclave, sealed, and kept in an oven at 220 °C for 72 h. After cooling to room temperature, the light green powder was harvested. The as-synthesized material was separated by centrifugation (x7000 for 10 min) and washed sequentially with water, methanol, and acetone. The washed sample was kept in DMF at 60 °C for 12 h. After the cleaning steps, the material was solvent-exchanged with methanol at ambient temperature for three days, refreshing the solvent three times a day.

**1.6. MIL-101(Cr) synthesis from commercial grade precursors**

Green powder MIL-101(Cr) was prepared in a similar hydrothermal procedure but by heating a mixture of Cr(NO_3_)_3_·9H_2_O (355 mg, 0.89 mmol), terephthalic acid (166 mg, 1.00 mmol), and deionized water (17 mL) at 180 °C for 48 h. The DMF-washed and methanol-exchanged sample was used for further studies.

**2. Characterization Techniques**

The prepared MOF samples in this study were characterized by Powder X-ray diffraction (PXRD), thermogravimetric analysis (TGA), ^1^H NMR spectroscopy, Fourier-transform infrared spectroscopy (FTIR), and low-pressure N_2_ and H_2_O adsorption measurements.

PXRD measurements were carried out at room temperature on a Bruker-AXS D8 powder diffractometer using Cu Kα radiation (λKα = 0.154186 nm) with a scan speed of 1.0° min^-1^ and a step size of 0.02° in 2θ.

TGA was performed under a continuous compressed air flow and recorded on a TA Instruments hi-res TGA Q500 thermogravimetric analyzer with a heating rate of 5 °C per minute.

The FTIR spectra of the synthesized samples were recorded on a Prestige-21, which uses a bright ceramic light source, high-sensitivity DLATGS (detector and high-throughput optical elements).

^1^H NMR spectrum was recorded on a Bruker Avance III 400 MHz instrument. Chemical shifts are reported in ppm (δ, relative to TMS) using DMSO residual peak (δ = 2.50 ppm) in DMSO-d_6_ as an internal standard.

SEM-EDS analysis were taken on a FEI Quattro S Environmental Scanning Electron Microscope (ThermoFischer). Samples were prepared by fixing the powder particles on a microscope holder.

A Flame Atomic Absorption Spectrophotometer (AAS PerkinElmer model Analyst 100) was used to measure the concentration of chromium (Cr) in the tannery effluent. The instrument readings were appropriately rounded off based on the standard deviation value from triplicate measurements. The wastewater sample used for Cr determination was collected in plastic bottles and was immediately acidified with 5 mL of concentrated HNO_3_ per liter of water. A 100 ml sample was transferred to a 250 ml beaker, and 5 ml of HNO_3_ was added. The sample was then evaporated to near dryness on a hot plate, cooled, and another 5 ml concentrated HNO_3_ was added, before returning to the hot plate and continuing to heat, adding additional acid as needed, until digestion was complete. The sample was then filtered into a 100 ml volumetric flask after being washed down the beaker walls with deionized water. The flask was then filled with deionized water to the mark. The prepared solution was used for the determination of Cr.

N_2_ adsorption measurements were performed on 3-Flex Surface Characterization Analyzer (Micromeritics) at relative pressures up to 1 bar. The cryogenic temperatures were controlled using liquid nitrogen baths at 77 K. The apparent surface areas were determined from the nitrogen adsorption isotherms collected at 77 K by applying the Brunauer-Emmett-Teller (BET) model. Pore size analyses were performed using DFT pore model systems. Prior to the sorption measurements, the as-synthesized MOF samples were washed several times with DMF and soaked in 10 mL of methanol for three days, during which time the methanol was exchanged three times per day. In a typical experiment, 100 mg of MOF sample was transferred (dry) to a glass sample cell, evacuated at 150 °C at a rate of 1 °C/min, held for 12 h, and cooled to room temperature.

**3. Supplementary Figures**





**Figure S1.** FTIR data for H_2_BDC obtained from the alkaline treatment of PET plastic bottles and its comparison with the FTIR spectrum of commercial terephthalic acid.


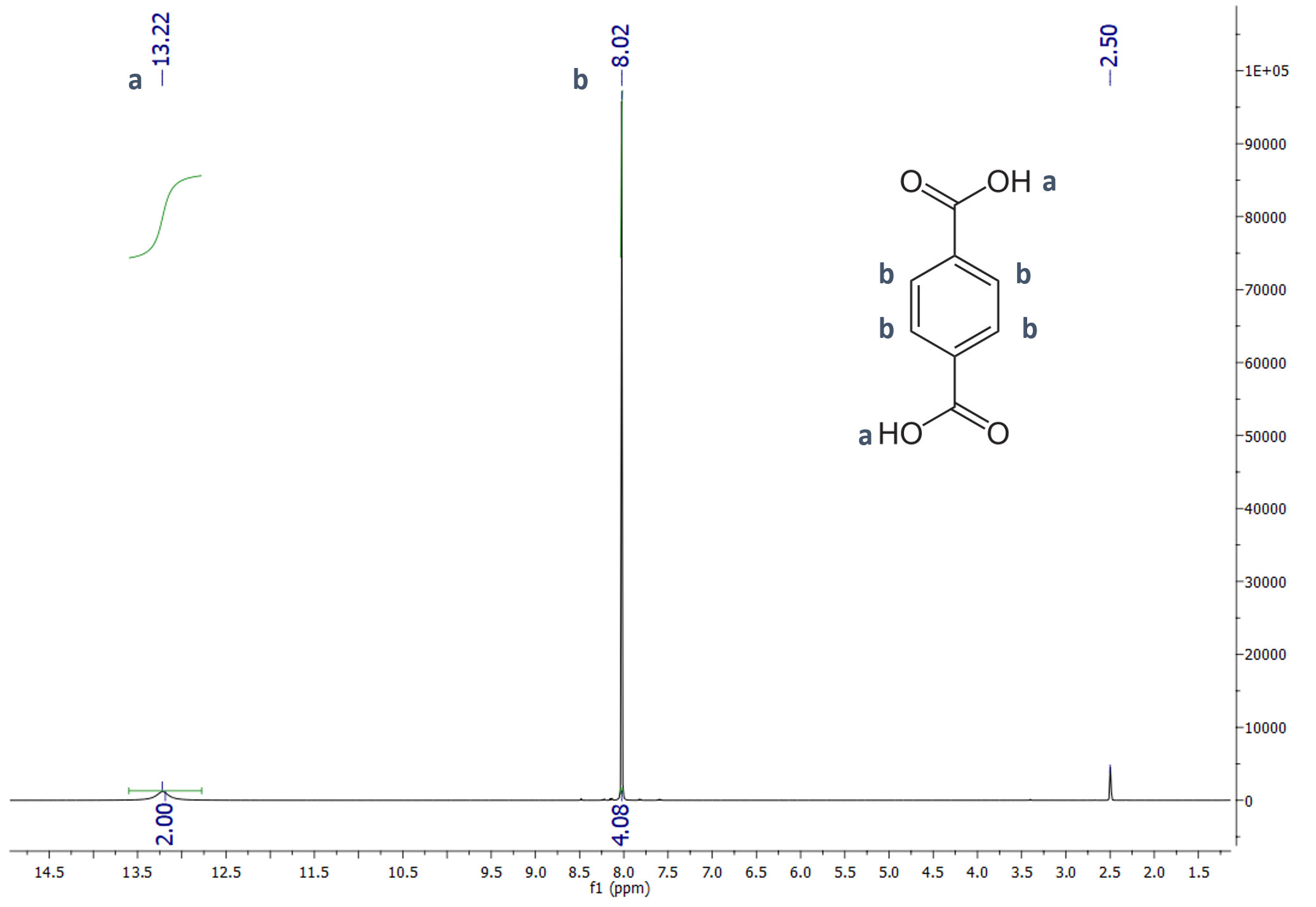


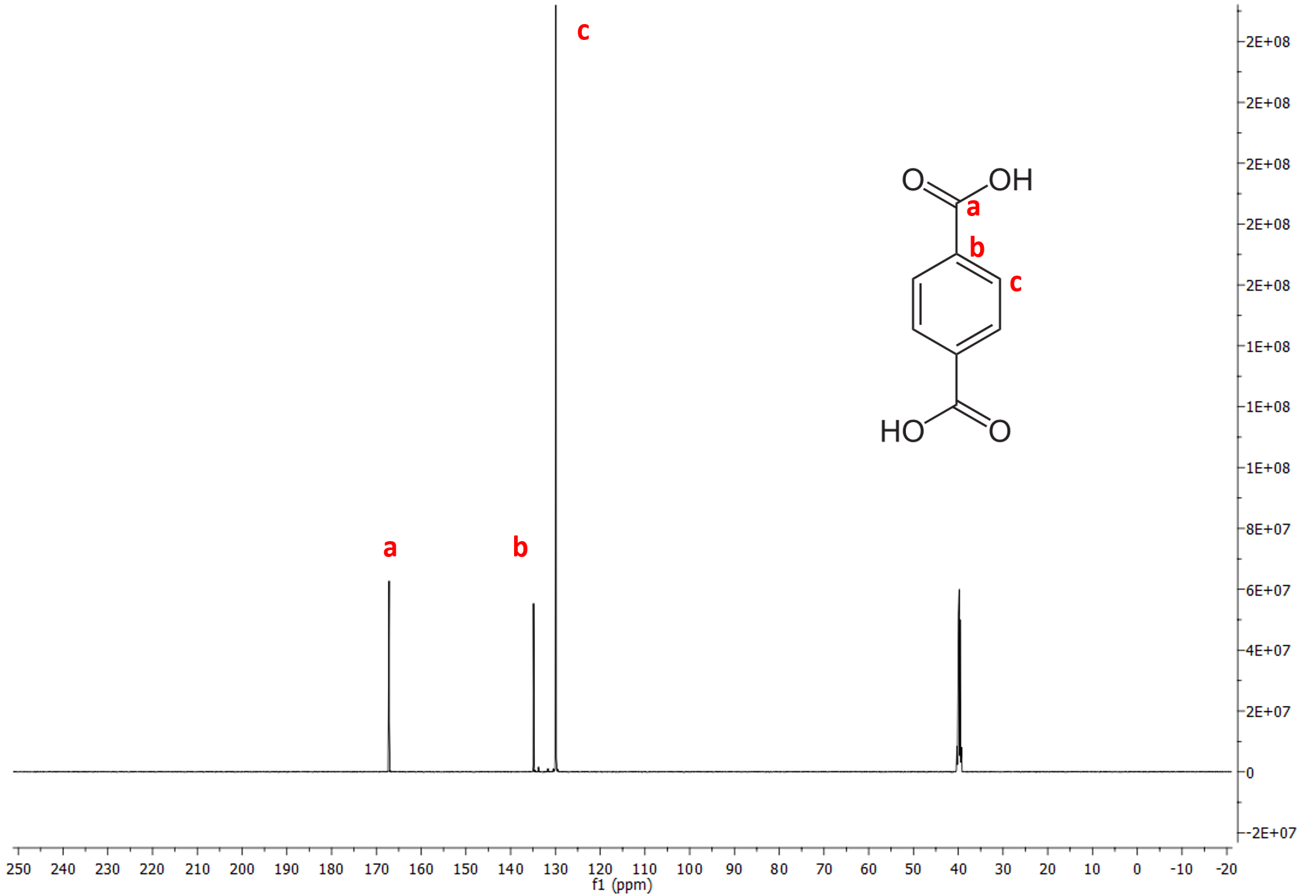


**Figure S2.** ^1^H NMR (Top) and ^13^C NMR (Bottom) spectra of terephthalic acid in DMSO-d_6_ obtained after alkaline treatment of PET plastic bottles.





**Figure S3.** Comparison of calculated and experimental PXRD patterns of MIL-53(Cr) obtained from commercial grade precursors and wastes.


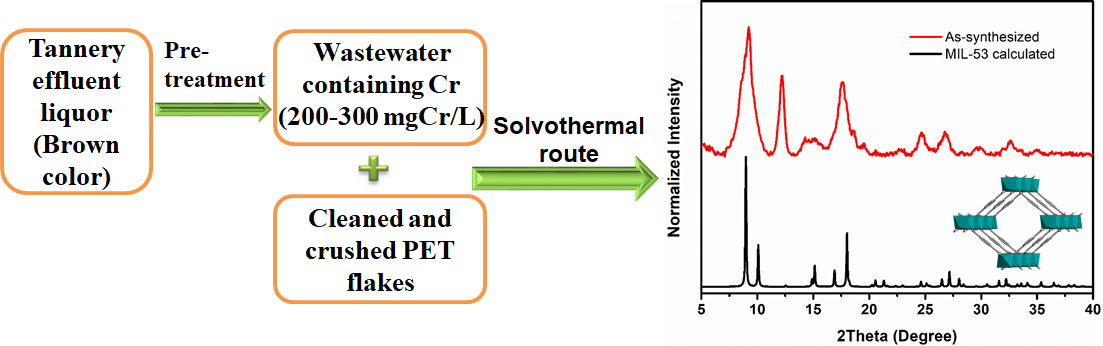
 **Figure S4.** Schematic showing the one-pot hydrothermal synthesis reaction to assemble MIL-53(Cr) from waste and comparison of the resultant experimental PXRD patterns with the calculated MIL-53(Cr) PXRD. The tannery effluent is pretreated by filtration to remove insoluble solid impurities (e.g., hair, fibers and other suspended substances), followed by evaporation to increase the chromium concentration by about tenfold.







**Figure S5.** PXRD diagrams of waste-derived MIL-53(Cr) and MIL-101(Cr) samples after soaking in water for three days.


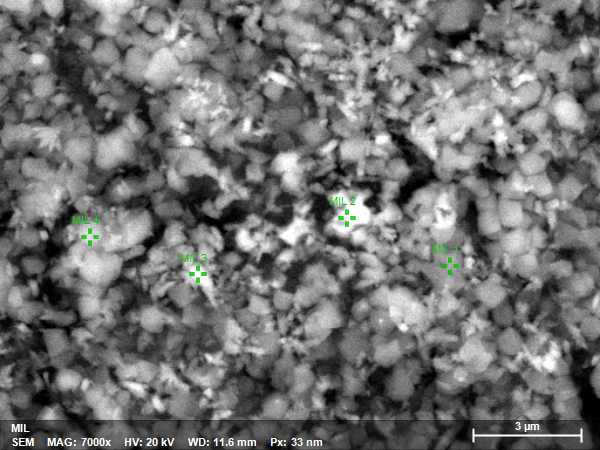


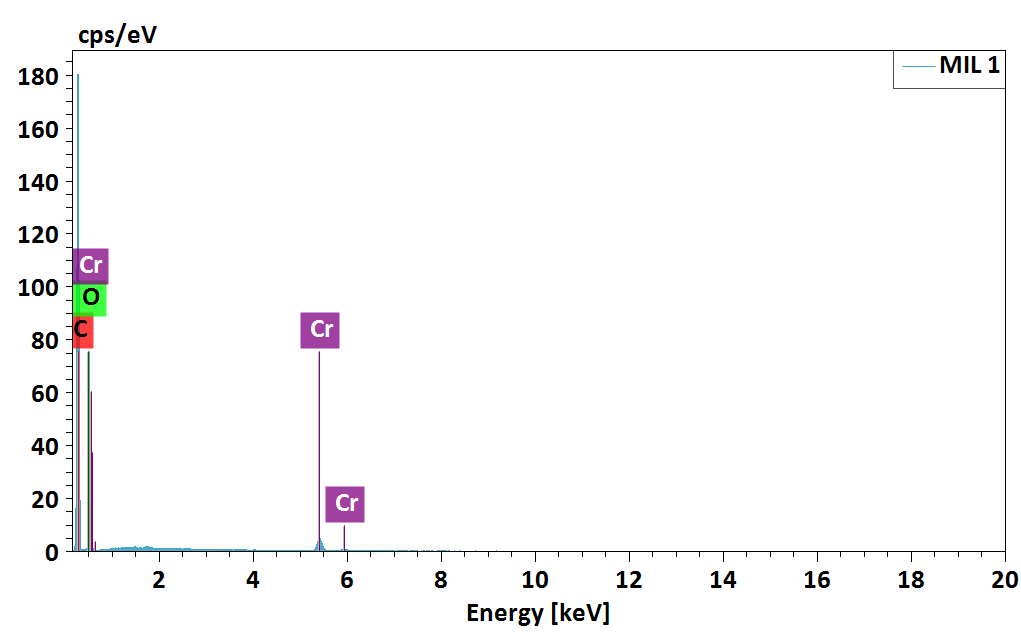


**Figure S6.** EDS analysis of the waste-based MIL-101(Cr) sample. The sample was degassed under vacuum overnight at 150 °C before the analysis.


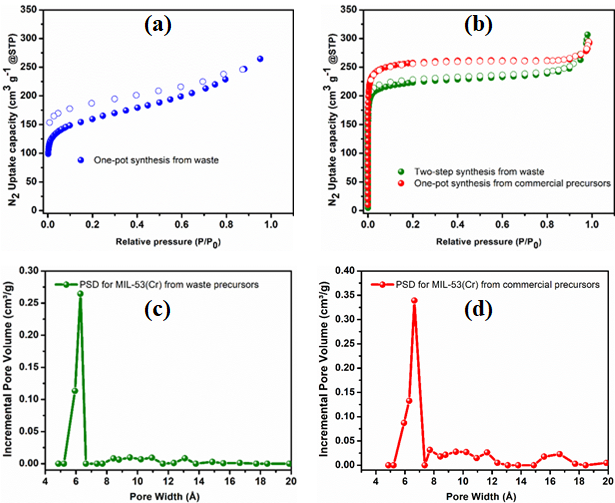


**Figure S7.** N_2_ sorption isotherms (closed symbols: adsorption and open symbols: desorption) collected at 77 K for MIL-53(Cr) synthesized by: (a) one-pot synthesis from waste; (b) two-step synthesis from waste and one-step synthesis from commercial Cr and H_2_BDC precursors; and pore size distribution analysis (DFT model) for MIL-53(Cr) assembled from (c) waste by the two-step synthesis and (d) commercial Cr and H_2_BDC precursors.

**References**

1 Xian, S., Lin, Y., Wang, H. & Li, J. Calcium-Based Metal–Organic Frameworks and Their Potential Applications. *Small* **17**, 2005165, doi:<https://doi.org/10.1002/smll.202005165> (2021).

2 Zhang, X. *et al.* Synthesis of acidic MIL-125 from plastic waste: Significant contribution of N orbital for efficient photocatalytic degradation of chlorobenzene and toluene. *Appl. Catal. B* **310**, 121300, doi:<https://doi.org/10.1016/j.apcatb.2022.121300> (2022).

3 Serre, C. *et al.* Very Large Breathing Effect in the First Nanoporous Chromium(III)-Based Solids: MIL-53 or CrIII(OH)·{O2C−C6H4−CO2}·{HO2C−C6H4−CO2H}x·H2Oy. *J. Am. Chem. Soc.* **124**, 13519-13526, doi:10.1021/ja0276974 (2002).
